# In Search of a Soil Moisture Content Simulation Model: Mechanistic and Data Mining Approach Based on TDR Method Results

**DOI:** 10.3390/s21206819

**Published:** 2021-10-14

**Authors:** Andrzej Brandyk, Bartosz Szeląg, Adam Kiczko, Marcin Krukowski, Adam Kozioł, Jerzy Piotrowski, Grzegorz Majewski

**Affiliations:** 1 Water Centre, Warsaw University of Life Sciences, 02-676 Warsaw, Poland; 2 Department of Geotechnics, Geomatics and Waste Management, Kielce University of Technology, 25-314 Kielce, Poland; bszelag@tu.kielce.pl; 3 Institute of Environmental Engineering, Warsaw University of Life Sciences, 02-787 Warsaw, Poland; adam_kiczko@sggw.edu.pl (A.K.); marcin_krukowski@sggw.edu.pl (M.K.); adam_koziol@sggw.edu.pl (A.K.); grzegorz_majewski@sggw.edu.pl (G.M.); 4 Department of Building Physics and Renewable Energy, Kielce University of Technology, 25-314 Kielce, Poland; piotrowski@tu.kielce.pl

**Keywords:** soil moisture content, dielectric permittivity, modeling, uncertainty, sensitivity analysis

## Abstract

Soil moisture content simulation models have continuously been an important research objective. In particular, the comparisons of the performance of different model types deserve proper attention. Therefore, the quality of selected physically-based and statistical models was analyzed utilizing the data from the Time Domain Reflectometry technique. An E-Test measurement system was applied with the reflectogram interpreted into soil volumetric moisture content by proper calibration equations. The gathered data facilitated to calibrate the physical model of Deardorff and establish parameters of: support vector machines, multivariate adaptive regression spline, and boosted trees model. The general likelihood uncertainty estimation revealed the sensitivity of individual model parameters. As it was assumed, a simple structure of statistical models was achieved but no direct physical interpretation of their parameters, contrary to a physically-based method. The TDR technique proved useful for the calibration of different soil moisture models and a satisfactory quality for their future exploitation.

## 1. Introduction

The importance of soil moisture as an environmental variable is evident from the viewpoint of its role in the hydrological cycle. Soil moisture impacts rainfall-runoff processes, infiltration, groundwater recharge rates, and constrains evapotranspiration as well as photosynthesis. To some extent, it governs water and energy exchange between the land, plants and the atmosphere [1,2,3,4], but embraces multiscale feedbacks as well [5]. The importance of soil moisture was stressed particularly in respect of the partitioning between the sensible and latent heat fluxes, and consequently for the temperature of the surface and the lower atmosphere [6,7]. From the viewpoint of mass and energy transfer, the storage and availability of water is crucial for plant growth, and its shortages are considered to be serious limitations to agricultural production. In recent years, the on-going climate changes have resulted in atmospheric and hydrological droughts that give rise to soil and physiological droughts affecting harvest yields and water supplies. The observed rise in air temperature along with precipitation shortages occur more frequently all over the world, e.g., in Europe and Poland, and affect the security of soil water resources and crop yields.

Since soil moisture content plays a significant role to satisfy water requirements not only of agricultural produce, but also in other numerous respects, the TDR method has become highly developed and applied worldwide for soil moisture estimation [8,9,10,11,12], being available for other materials as well [13,14,15]. That method involves electrical permittivity as an indirect parameter of soil moisture content estimation. Electrical permittivity ε [−] is a measure of matter molecule behavior in the case of external, variable electrical field application [16]. The values of that parameter for individual phases of porous media are the following: air—1 [−], soil solids 1–15 [−], water—80 [−]. The occurring difference between the permittivities of individual phases is the base for dielectrical methods application, including TDR.

Based on the data from the TDR method, the knowledge on temporal variability of soil moisture content in different hydrological conditions, in particular, becomes indispensable for producing environmental forecasts and improving their predictions [17]. It is also of significance to a number of applications, e.g., management practices that may currently employ soil moisture data all over the world. This is also the basis to acknowledge crop plant growth issues but also an effort to better understand and predict soil water storage in short time increments, e.g., on a daily basis.

In this work, we focused on the performance of various models for soil moisture content simulation, taking advantage of the measurement data obtained by the TDR method. In addition to the physically-based models, the literature review [18] shows that potential is offered also by data mining methods [19]. They facilitate finding relatively simple models that involve easily obtainable hydrometeorological variables, such as air relative humidity and temperature, precipitation, or others, in order to simulate the moisture content of the shallow soil layers.

Among all relevant models, artificial neural networks, support vector machines, Random Forests, boosted trees, and k-nearest neighbor should be included [20]. In these methods, at the training stage, the model structure is formulated based on historical data on different input variables. This becomes decisive for the obtainable quality of model simulations, which is usually indicated by the values of mean absolute and relative errors. The main advantage is the non-complex and flexible model structure which was was attempted in this work for the selected statistical and physical models. Additionally, the volumetric soil water contents, recorded by a selected type of sensor, were studied to prove their reliability for the calibration of different soil moisture mathematical models [21]. 

## 2. Materials and Methods

**Figure 1:**
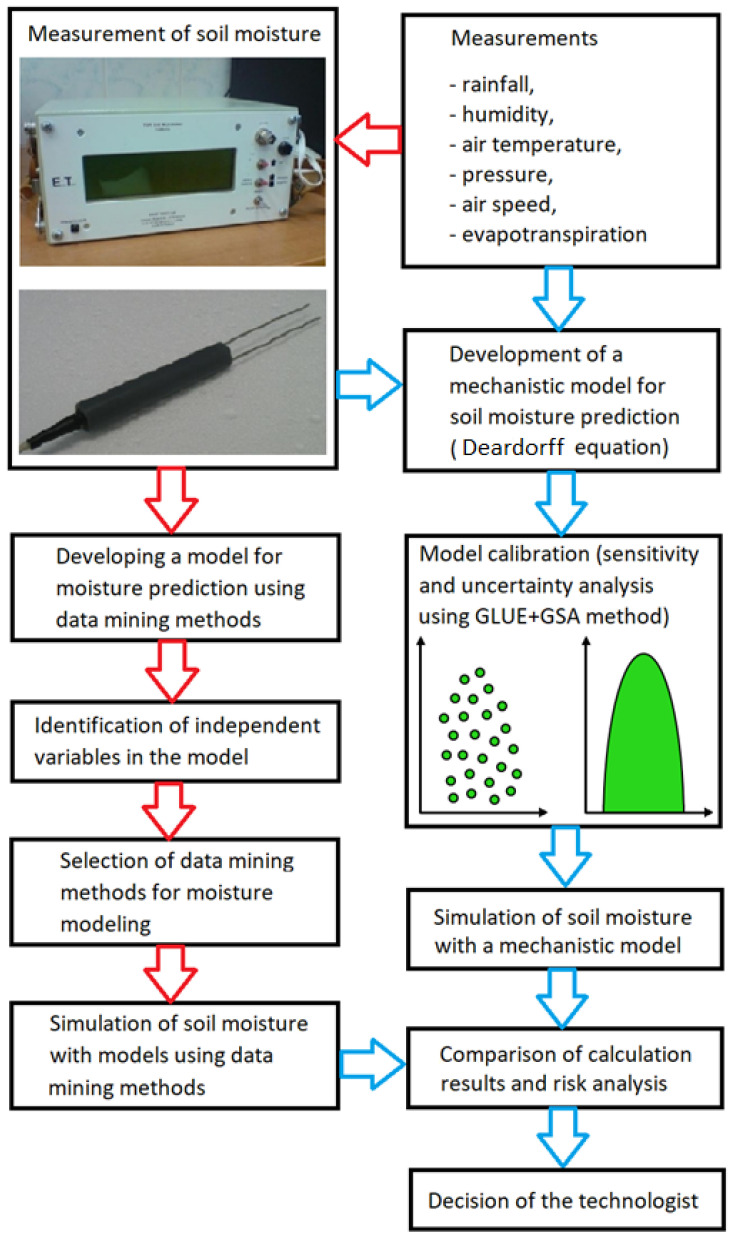
Algorithm for soil moisture model determination.

The analyses involved an algorithm for the selection of the soil moisture content forecast model. Two mathematical models were taken into consideration: a statistical one and a physical model by Deardorff [6,22] which was especially developed and dedicated for shallow soil layers moisture, shaped by daily weather variations. The comparison of the models is a base for their further exploitation in the field of water resources management and related decision-making. The sense of comparative studies and resultant model selection would facilitate to approach either water shortages or excess (i.e., drought or flood), and to mitigate environmental and ecological threats (not overlooking economic issues). With reference to the above-mentioned problems, an appropriate scheme was proposed (Figure 1).

The considerations involved the observation data included: precipitation, air relative humidity, and air temperature and wind speed, to elaborate soil moisture forecast models through data mining methods. The calculated potential evapotranspiration and precipitation were input to a physical model.

The main advantage of statistical methods, in comparison to the physical approach, is a much more simple model structure. In a statistical model, the independent variables which significantly influence the phenomenon are distinguished in the first instance. From a practical point of view, a compromise is established between the model complexity, usefulness, and exploitation possibilities. Hence, the analyses performed herein embraced a couple of data mining methods so that the proper model for soil moisture forecast could be found. In reference to the statistical model, the creation of the physical model (mechanistic one) seems to be more complicated. This is due to variability of weather and soil conditions as described by a number of relevant equations [23,24], and the one by Deardorff in particular, which requires the employment of numerical methods as well as proper differential schemes to be solved. Owing to the existence of considerable interactions between the coefficients of the Deardorff equation there arise problems in their identification that hampers its usefulness.

In order to acknowledge existing constraints in the identification of Deardorff equation parameters a probabilistic estimation was realized through coupled General Likelihood Uncertainty Estimation (GLUE) and Global Sensitivity Analysis (GSA) methodology [25]. Such an approach facilitated to reach reliable distributions of the calibrated parameters in the Deardorff equation, that governed the range of simulation results (95% confidence level).

Based on the determined models (a physical and a statistical one), the simulations of the soil moisture content were performed for the adopted input values. The aforementioned expected model accuracy, resulting from measurement data, would delineate the aftermath of the latter decisions regarding water storage, testing of irrigation efficiency or drainage and irrigation practices, for instance. It would take effect further in evaluation of ecosystem services or ecosystem potential, bringing about uncertainties in the decision-making process.

### 2.1. Measurements of Meteorological Parameters

**Figure 2:**
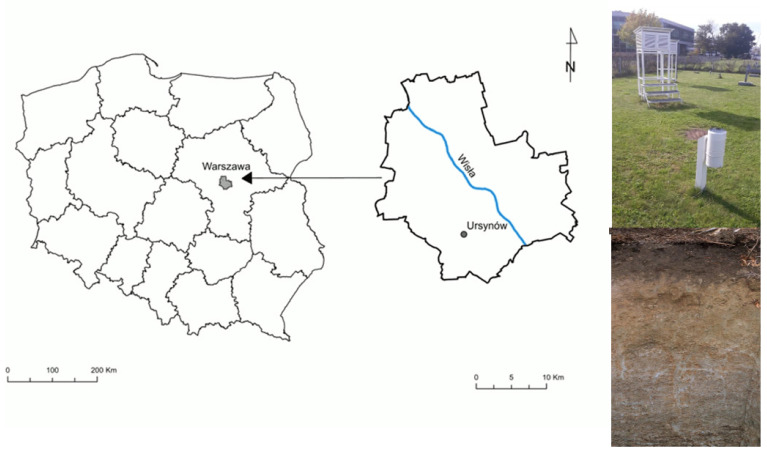
Location of the research site.

The research was performed for the southern district of Warsaw (Ursynów district) within the compounds of Warsaw University of Life Sciences (Figure 2). That area is characterized by a high anthropopressure degree and a high coverage by impervious areas, e.g., the presence of large areas of housing, sidewalks, car parking lots and main streets, that contribute to a fast generation of overland flow.

However, the percentage of permeable surface includes city parks, and extensive green and recreational areas, which causes water storage in the soil to be essential for the sake of city adaptation to a changing climate. This means there is a need for proper models for the soil moisture content that employ easily obtainable hydrometeorological variables (Table 1). In this respect, the indispensable data range originated from the WULS Ursynów meteorological station (λE 21°02′52″ φN 52°09′38″, 102.5 m a.s.l.) was used. 

Daily precipitation acted as model input for soil moisture simulations, as well as air temperature, relative humidity, and windspeed, that became also indispensable for potential evapotranspiration estimates according to FAO procedure [26] on hourly time step by the Penman–Monteith equation:

(1) $$ET_{0}=\frac{0.408\cdot \left(R_{n}-G\right)+\gamma \cdot \frac{900}{T+273}\cdot u_{2}\cdot \left(e_{s}-e_{a}\right)}{∆+\gamma \cdot \left(1+0.34\cdot u_{2}\right)}$$

where *ET_o_* is reference evapotranspiration [mm day^−1^], *R_n_* is net radiation at the crop surface [MJ m^−2^ day^−1^], *G* is soil heat flux [MJ m^−2^ day^−1^], *T* is air temperature at 2 m height [°C], u_2_ is wind speed at 2 m height [m s^−1^], *e_s_* is saturation vapor pressure [kPa], *e_a_* is actual vapor pressure [kPa], *e_s_* is *e_a_* saturation vapor pressure deficit [kPa], Δ is slope of vapor pressure curve [kPa °C^−1^], and *γ* is psychrometric constant [kPa °C^−1^]. Hourly evapotranspiration data were then averaged to obtain mean daily evapotranspiration.

### 2.2. Measurements of Soil Moisture Content

The selected research site within the WULS campus (the meteorological station) was subject to measurements of the volumetric soil moisture content by the TDR method. The geological setting of that site exhibited generally sandy loams up to 1.9 m depth [27] underlain by sandy clay higher than 10 m in thickness, along with deep ground water table, reaching 11 m beneath the land elevation. The conducted survey revealed soil layers to the depth of 0.2 m, for which moisture was monitored by TDR probes, containing 65% of sand (diameter 2–0.05 mm, including: 24% of coarse sand (1–0.5mm), 16% of medium (0.5–0.25 mm), 13% of fine sand (0.25–0.1 mm) and 12% of very fine sand (0.1–0.05 mm)). The estimated fraction of silt equaled 24% (diam. 0.05–0.002 mm), along with 11% of coarse silt (0.05–0.02 mm) and 13% of fine silt (0.02–0.002 mm), and finally the fraction of clay (diam. < 0.002 mm), reaching 11% [28]. Soil particle size composition was made available by laboratory sieves, that offered diameters from 1 mm to 0.05 mm. However, silt and clay fractions were found through hydrometer (areometric) analyses [29].

Field Operated Multimeter (FOM, E-Test, Lublin, Poland) was used for the soil moisture content measurements, equipped with electromagnetic impulse generator of a falling and rising time equal to about 3 × 10^−10^ s (300 ps). The generated impulse propagated along the concentric cable (6 m long) of a constant and invariable dielectric permittivity [30]. Then, the impedance changes in the cable–probe system caused the signal reflections, that were next used to determine the time of propagation along the measurement rods (100 mm long, parallel, stainless, 2 mm in diameter with a spacing of 16 mm). The first reflection (the initial one) took place at the cable–probe interface, and the second occurred at the end of the probe. Those reflections were intercepted by the FOM meter in the form of a so-called reflectogram, and automatically interpreted into electrical permittivity and then into soil volumetric moisture content with the use of calibration equation by Malicki [31]. The reliability of the applied equation was proved through comparisons to gravimetric method for sixty soils all over the area of Poland for a range of soil densities. Determination coefficient for the compared variables was equal to 0.98 and the absolute, average error of moisture content reached the value of 3%. The TDR probes, such as FP/mts, were applied here, that also made it possible to record the soil temperature by AD592CN sensor. They facilitated the measurements performed at two soil depths: 10 and 20 cm at the abovementioned meteorological station, where the land surface was covered by standard, mown grass of 10 cm height. All data were recorded for vegetation periods (01.04–30.09) of 2011, 2013 and 2014.

### 2.3. Data Mining Methods for Soil Moisture Content Simulation

A literature search [32] revealed a range of data mining methods that can be applied for the sake of environmental processes simulation. An advantage is the simple structure of the models and relatively small number of parameters or coefficients that require identification in a proper manner. On the other hand, a number of cases are thought to employ models of a complex structure with numerous parameters subject to calibration. In principle, more complex models should provide better match of the modeled and observed variables, i.e., higher accuracy. It should be stressed, however, there is no actual rule, since many cases proved relatively simple models to offer high or acceptable accuracy of the results not very different from the complex ones.

Modeling of processes occurring in the natural environment frequently employs multivariate regression methods; however, there exists linear relationships between dependent and independent variables that are found to restrict application possibilities. In response to existing limitations, the required refinements led to MARS (Multivariate Adaptive Regression Spline) determination. The main advantages of MARS lie in its capacity to capture the intrinsic, complicated data mapping in high-dimensional data patterns and produce simpler, easier-to-interpret models, as well as its ability to perform analysis on parameter relative significance. In addition to the complete involvement of the predictors in MARS procedure, there are also all observations of the independent variable being analyzed. Moreover, their variability range becomes divided into intervals where the interval limits are derived from the so-called knots, which, in turn, constitute the threshold values (t) [33]. In this manner, by comparing the values of the independent variable ($x_{m}$) with the threshold ones ($t_{m}$), the sign of the relationship y = f(x_i_) can be determined. The threshold values are utilized to distinguish the values of the independent variable assuming the base functions such as $\;\beta_{i}\cdot \max\;\left(0,X-t\right)$. The general form of the MARS model is achieved through summing the base functions and their products as follows:

(2) $$y=\beta_{0}+\overset{M}{\underset{m=1}{\sum }}\beta_{m}\cdot H_{k,m}\left(X_{\upsilon \left(k,m\right)}\right)$$

(3) $$H_{k,m}=\prod_{k=1}^{K}h_{km}$$

where *k* is the number of independent variables, *M* is the number of components of the created model, *m* = 1, 2, 3, …, *M*, and *β_m_* is the weight values.

In order to acknowledge the fact of measurement data falling into clusters, for instance, the procedure for data partitioning was identified through proper rules dependent on the values of independent variables. That finding was successfully implemented in the method of regression and classification trees. However, due to emerging problems with those models’ stability, the modification was imposed to replace a single tree with the forest (parallel connected trees—Random Forest) or a serial layout (boosted trees algorithm). The adopted solutions provided better predictive capabilities of the regression tree. Both Random Forest and boosted trees structure require identification of the number of trees, which should not be higher than 300 in practice [34]. On the other hand, an alternative approach to MARS, RF and BT is offered by the neural network method (ANN), in which model structure and the number of estimated coefficients is higher than in the above-mentioned models. Nonetheless, one of the frequently applied ANN models is the multilayer perceptron (MLP) that consists of three layers: an entrance layer, a so-called hidden layer, and the output layer. The neurons of the individual layers are connected by means of proper weights, which are estimated at the stage of model learning. Because of the significant influence of initial values of the weights on the simulation results, and also problems with determining the global minimum of the analyzed functional relationships, a proper modification of MLP was introduced. If no amendments were introduced, possible limitations to the predictive capabilities of that model may occur, which cause the results of MARS, BF and BT to become comparable or even more precise than the ones achieved by MLP. In the method of SVM (support vectors method) the shortcoming of the neural networks (MLP) was eliminated by replacing the so-called hidden neurons with Kernel functions that facilitated to define the conditions of a global minimum occurrence. The above modification considerably influenced the predictive capabilities of SVM in comparison to MLP. However, in particular the SVM requires the identification of both: the insensitivity threshold (*ε*) and capacity (C) for the coefficients of namely the Kernel core (*α_i_*), stressing that the cases of limited data quantity may affect the optimum values of the coefficients, and finally diminish the predictive capabilities of the model.

The determination of MLP models involved the number of neurons within the range from j to 2j + 1. In order to optimize the model structure for the adopted number of neurons, there was the need to analyze the proper, so-called activation functions in the hidden and the output layer (assuming particular function types: linear, exponential). Neuron number and activation functions were determined through consecutive iterations, leading to initial model structure. In the MARS method, the recursive algorithm was implemented both for model building and for independent variables selection, while SVM adopted the Gaussian form of Kernel functions, and the values of C, ε, and α were obtained through consecutive substitutions method. The GP (genetic programming) models were derived from mathematic operators such as +, −, /, and basically relied on the initial number of individuals *n* = 200, the generations *N* = 300, with the assumed mutation probability *P_m_* = 0.25 and the crossing probability *P_c_* at the level of 0.65.

### 2.4. Deardorff Equation

In order to describe relationships between meteorological parameters and soil moisture, the quasi- physical model was utilized, based on the concept presented by Deardorff [6]. The choice of that methodology was conditioned by simplicity aspects and appropriateness to represent diurnal variation influence on water regime of the shallow soil system. According to original assumptions, the variation of near-surface volumetric soil water content can be approached through mass exchange between the atmosphere and two distinguished soil layers: upper and lower. The first one, namely the surface layer, constitutes the transitional zone of the soil moisture controlled by daily weather variations to a large extent. Its thickness is assumed to be 10 cm. In the second layer, the moisture content changes seem to be visibly lower. The Deardorff model incorporates then only two time-dependent variables: the soil-surficial moisture content, for a first layer, and the bulk moisture content of the second one:

(4) $$\frac{\partial w_{1}}{\partial t}=-C_{1}\cdot \frac{E_{g}-P}{h_{1}}-C_{2}\cdot \frac{w_{1}-w_{2}}{\tau }\;,\;0<w_{1}\leq w_{max}$$

(5) $$\frac{\partial w_{1}}{\partial t}=-C_{1}\cdot \frac{E_{g}-P}{h_{1}}-C_{2}\cdot \frac{w_{1}-w_{2}}{\tau }\;,\;0<w_{1}\leq w_{max}$$

where *w*_1_, *w*_2_ denotes the soil water content (–) at upper and lower layer, respectively, *h*_1_ and *h*_2_ are layer thickness (m), *P* is precipitation rate (m/s), *τ* is diurnal time period (1 day expressed in seconds), *C*_1_, *C*_2_ are dimensionless proportionality coefficients, *E_g_* is the bare-soil evaporation (m/s), *μ* is the constant used to account for the moisture transport below a lower layer and *w_max_* is the soil field capacity. The *C*_1_ and *C*_2_ parameter values rely on the soil moisture and texture as well as the functional relationships reported by Noilhan and Planton [35]. In the Deardorff approach the *C_2_* parameter is treated as a constant and *C_1_* becomes a piecewise function of effective saturation (*C*_1_ = f(*w*_1_*/w_max_*)). The present model follows the original Deardorff formulation; however, the authors adopted another type of function for *C*_1_. Eventually, its value is derived against effective saturation, but as a power function of a single parameter:

(6) $$C_{1}={\left(\frac{w_{1}}{w_{max}}\right)}^{m}$$

where *m* denotes the model parameter. That modification was imposed to reduce the number of parameters, as a piecewise, original relationship requires two more. Assuming *m* < 0, the proposed equation preserved the decreasing character of the *C*_1_ coefficient with saturation. The exponential form of that equation also showed better agreement with an experimental set proposed by Noilhan and Planton [35]. With Equation (5), the dependency given with the data points for all three soil classes, sand, loam and clay, was successfully reproduced, and the coefficient of a linear correlation became higher than 0.95.

The soil moisture transport was not involved in the Deardorff model as it was limited to a time period of a few weeks only. Here, to extend the time scale, the sink term was added to Equation (4), that permitted taking account of the moisture transport outside the model’s domain. That term was utilized as linear relationship, applied before in the similar studies [36]. The bare-soil evaporation rate (*Eg*) was linked to the potential evaporation (*Ep*) with the following Equation (7):

(7) $$E_{g}=\left\{\begin{array}{c} \left(\frac{w_{1}}{w_{sat}}\right)\cdot E_{p}\;w_{1}<w_{s}\\ E_{p}\;\;w_{1}\geq w_{s}\; \end{array}\right.$$

where *w_s_* ≈ 0.75 *w_max_*.

The solutions of Equations (4) and (5) were achieved through explicit Runge–Kutta procedure, offered by the ode23 solver as the component of the Matlab computation software [37]. The variables *m*, *C*_2_, *μ* and *w_max_* were considered to be model parameters.

### 2.5. Coupled GSA-GLUE Methodology

The basics of the GSA-GLUE technique lie in the probabilistic identification of parameters according to original GLUE framework. A common practice is to search for one deterministic parameter set that provides the closest match of the model-simulated and measured values. Probabilistic approach facilitates the case of models with a considerable number of parameters since the inverse modeling may lead here to many equivalent solutions. The existence of different parameter combinations, that allow to sufficiently represent the process dynamics, results from lack of reliable data that provide ambiguous parameter values. Therefore, the solution is offered by adopting probabilistic parameter identification. In this case the existence of only one optimum combination of parameter values is dismissed on account of random distribution, which properties are to be determined in the model identification process. Probabilistic description of parameter space in the GLUE method provides also for the description of model uncertainty itself, being attributable to insufficient data number, but also imperfect representation of the real process (e.g., inaccurate data, inadequate mathematical description, simplifying assumptions) [38]. The problem of GLUE parameter estimation was formulated as Bayesian analysis:

(8) $$M\left(X|w\right)=\frac{M\left(X\right)M\left(w|X\right)}{M\left(w\right)}$$

where X is the vector of model parameters $\left[m\;C\;\mu \;w_{max}\right]$, $M\left(X\right)$ is a priori distribution of parameters, w is the vector of measured soil moisture contents, $M\left(w|X\right)$ is the reliability function, $M\left(X|w\right)$ is resultant distribution a posteriori, and $M\left(w\right)$ is the scaling factor (such that $\Sigma M\left(w|X\right)\;=1$).

A priori distribution $M\left(X\right)$ reflects initial assumptions on parameters’ variability. In the case of models of surface runoff either soil moisture content or the structure of the parameters’ distribution is not known, but only their range or allowable limits resulting from their physical interpretation. A constant character of the parameter distribution was adopted for soil moisture content modeling herein, which is a frequent case in such or similar applications [38]. Moreover, GLUE parameter identification embraced the transformation of a priori distribution to a posteriori one through reliability function as conditioning the probability of parameter combination due to the fitting quality of the modeled and measured values. In the analyses herein the following form of the reliability function was applied:

(9) $$M\left(X|w\right)=p\cdot exp\left(\frac{-r}{k\cdot V\left(r\right)}\right)$$

where *p* is scaling parameter, k is a shape factor used to control a variance of function value, and *V(∙)* is the variance r-averaged squared deviation of modeled and simulated values:

(10) $$r=\frac{1}{N}\cdot \overset{N}{\underset{k=1}{\sum }}{\left(w_{m,k}-w_{s,k}\right)}^{2}$$

where *w_m,k_* is the measured soil moisture content, and *w_s,k_* is the simulated soil moisture content.

According to GLUE procedure assumptions Equation (8) is solved by means of the Monte Carlo method. In the first stage, the parameter sample is created based on the adopted a priori distribution. Then, the model is launched for either combination of parameters, that forms the following vector, *X* = [*x*_1_, *x*_2_,..., *x_i_*], and based on the calculated and measured soil moisture contents the reliability function values (*w*) and resultant a posteriori distribution of parameters are determined.

The supplementary element to the GLUE method was introduced as GSA (global sensitivity analysis). It facilitated to quantify the impact of parameter variance on the variance of model results, and finally to distinguish those parameters that significantly influence the model uncertainty. GSA belongs to variance-based methods and relies on variance decomposition, as follows:

(11) $$V\left(Y\right)=V\left[E/X_{i}=x_{i}^{*}\right]+E\left[V\left(Y/X_{i}=x_{i}^{*}\right)\right]$$

where *Y* is the model output and *E*(*Y*|*X_i_* = x_i_*) and *V*(*Y*|*X_i_* = x_i_*) is the model expected value and variance, respectively, for the *i*-th parameter *X_i_* to x_i_*. 

The functions *E*(∙) and *V*(∙) are defined for *Xi* parameter that takes different values in its variability range. Once a particular sensitive parameter has been revealed, the output variance *E*[*V*(*Y*|*X_i_* = x_i_*)] takes relatively small values for different *X_i_* since the component *V*(*Y*|*X_i_* = x_i_*) does not involve the variability of that sensitive parameter. The significance of a parameter, on the other hand, will be manifested through high variance values of *V*[*E*|*X_i_* = x_i_*] on the condition that *X_i_* parameter changes along with output expected value. The model output variance is described by the second component of Equation (11) and is related with the direct impact of the *X_i_* parameter variance. That was the base to propose the model sensitivity measure to *i*-th parameter in GSA procedure, the so-called first order sensitivity indicator (S_i_):

(12) $$S_{i}=\frac{E\left[V\left(Y/X_{i}=x_{i}^{*}\right)\right]}{V\left(Y\right)}$$

Variance decomposition according to Equation (11) can be executed against all other parameters, (*X_–i_ = x_–_*_i_*) than only on *X_i_*, as follows:

(13) $$V\left(Y\right)=V\left[E/X_{-i}=x_{-i}\right]+E\left[V\left(Y/X_{-i}=x_{-i}\right)\right]$$

In Equation (13), in the same manner as (11), the first component describes part of the model variance, being a direct effect of the parameters *X_–i_* on the model output *Y*. The second component, however, represents the influence of *Xi* parameter being in interaction with other parameters (*X_–i_*) [38]. On this basis, the total sensitivity index is defined (*S_Ti_*) for the model against *X_i_* parameter as follows:

(14) $$S_{Ti}=\frac{E\left[V\left(Y/X_{i}=x_{i}^{*}\right)\right]}{V\left(Y\right)}$$

The general problem of sensitivity indexes determination by Equations (12) and (14) is the necessity to know the variance of the parameters. A solution is offered by the coupled sensitivity analysis and parameter identification in GSA-GLUE. Sensitivity analysis is performed on the index that takes the same form as the reliability function in the GLUE method and enables to analyze the model variance as identified with the uncertainty herein. Variance decomposition is realized by means of Monte Carlo technique with the adoption of proper structure of a sample to determine the values of parameter ensemble *X_i_* = x_i_* and *X_–i_* = *x_–i_*, according to Equations (11) and (13). Most frequently, the properly prepared sample is utilized both for GLUE parameter identification and sensitivity analysis.

In this paper the probabilistic identification of Deardorff model parameters *m*, *C*_2_, *μ*, and *w_max_* was accomplished by coupled GLUE-GSA methodology. Constant (a priori) distributions of the parameters were assumed according to literature data [24]. Parameter ranges are given in Table 2, and based on their values as well as assumed distributions the coupled GLUE-GSA analyses were performed aiming at the estimation of first order sensitivity indexes (S_i_) as well as total sensitivity (S_Ti_).

Based on the above-mentioned analyses, a multidimensional distribution of the parameters *m*, *C*_2_, *μ*, and *w_max_* were determined so as to describe the reliability of the Deardorff model. That model was calibrated on soil moisture content data for the first vegetation season (first period) and validated on the available dataset for the second and third period, so as to test the accuracy of the model in different meteorological conditions.

### 2.6. Aspects of Soil Moisture Content Estimation

Since soil moisture content may play a role as an indicator in decision-making processes (e.g., catchment management planning, water resources distribution and allocation) the offered accuracy of the relevant, applied mathematical model seems to be crucial. Taking final decisions is directly dependent on the achieved simulation results (*y_mod_*).

The measure of the decisions’ reliability and their potential effects can be expressed as follows:

(15) $$\left\{\begin{array}{c} Rs\left(t\right)=y_{mes}\left(t\right)-y_{mod}\left(t\right)\\ Rs{\left(t\right)}_{d}<0\\ RS{\left(t\right)}_{g}>0 \end{array}\right.$$

where *y_mes_* is measured soil moisture content, *y_mod_* is modeled soil moisture content, and *Rs*(*t*) is a function of either potential ecological or economic effects based on discrepancies between the modeled and observed variables (data). The *Rs*(*t*) function takes different values depending on meteorological conditions (e.g., the season of the year, vegetation period, drought, etc.).

In exploitation practice two cases may occur, whether the modeled values are higher than measured ones or vice versa: *Rs*(*t*)*_d_* < 0 and *Rs*(*t*)*_g_* > 0, respectively. In the first case, the decision-making needs to acknowledge that the managed amount of water is actually lower than the theoretical, calculated one (*y_mod_*). This can be a constraint on, e.g., drainage–irrigation scheduling or other solutions (retention, temporary water damming) in order to maintain a required soil moisture content. As a consequence, the environmental or ecological problems may occur related with the nature of the analyzed catchment and the discussed, relevant management issues. The value of *Rs*(*t*)*_g_* > 0 indicates additional soil water reserves that were not taken into account in the calculation phase. However, in this case the foreseen water requirements in the catchment can be treated as satisfied. Existing water excess, on the other hand, could be subject to reservoir retention or energy production. Whatever the solution, usually dependent on the existing infrastructure status, the proper maintenance of catchment resources would require modifying Equation (15) as follows:

(16) $$\left\{\begin{array}{c} Rs\left(t\right)=y_{mes}\left(t\right)-y_{mod}\left(t,\theta \right)\\ Rs{\left(t,\theta \right)}_{d,g}\to min\\ \theta =f\left(\varkappa ,\;x_{1},\;x_{2},\;\ldots ,\;x_{j}\right) \end{array}\right.$$

where *θ* is the function for the simulations of the soil moisture content dependent on meteorological conditions (in the situation of a deep groundwater table). Two models were adopted here: a mechanistic one, 𝜘 = 1, and a statistical one, 𝜘 = 2, which are chosen to execute the simulation due to prevailing local conditions in such a manner that the available expert knowledge helps to minimize the differences between the model and observations.

At last, the analyses of predictive capabilities of the models were performed in order to reveal the periods of simulation results over- or underestimation, or if the measured data shows considerable mismatch against the modeled variables. In order to achieve that aim the so-called “heat maps” were prepared so as to classify modeling errors on a properly assumed scale.

## 3. Results and Discussion

### 3.1. Coupled GLUE-GSA Method

**Figure 3:**
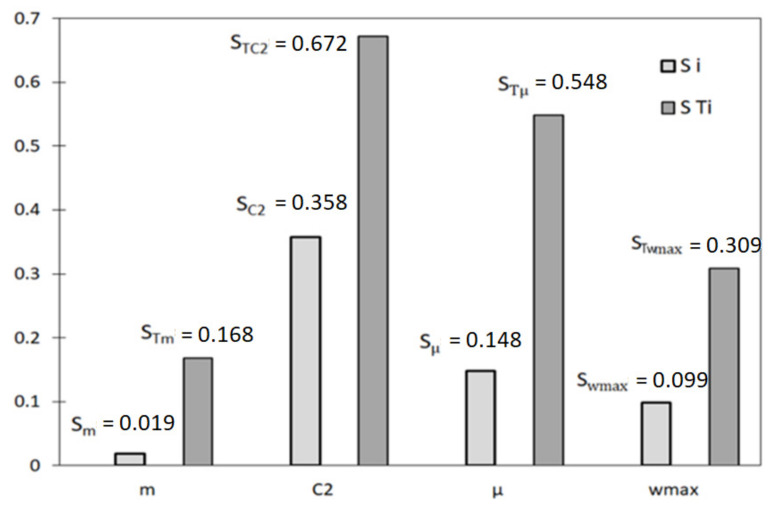
First order (S_i_) and total (S_Ti_) sensitivities for the analyzed parameters (*m*, *C*_2_, *μ*, *w_max_*) of Deardorff equation.

**Figure 4:**
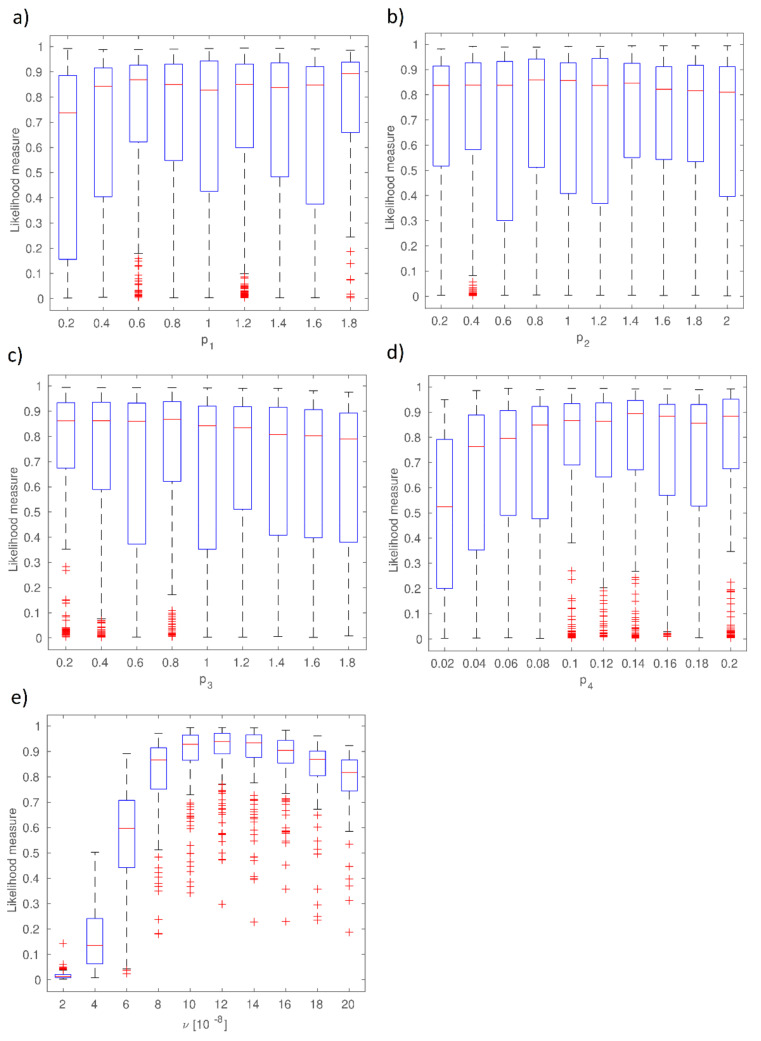
Reliability functions (p_1_, p_2_, p_3_, p_4_, υ) for the calibrated parameters of Deardorff model: (**a**) reliability of *C*_2_ parameter, (**b**) reliability of *m* parameter, (**c**) reliability of *μ* parameter, (**d**) reliability of *w_max_*, (**e**) model reliability function.

Based on (*C*_2_, *m*, *μ*, *w_max_*) parameter variability ranges the simulations of their numerical values were performed by means of Monte Carlo procedure in the first instance. Obtained vectors (*C*_2_, *m*, *μ*, *w_max_*) for the measured values (the values of precipitation and evapotranspiration) were incorporated into the Deardorff equation and then the probabilistic solution of the soil moisture content *w*_1_ and *w*_2_ was achieved. Taking into consideration the measurement data and the results achieved through Equations (11) and (13), the first order (S_i_) and total (S_Ti_) sensitivities were calculated (Figure 3). At the same time, the reliability functions for the consecutive, calibrated parameters of the Deardorff equation were determined and are given in Figure 4.

**Figure 5:**
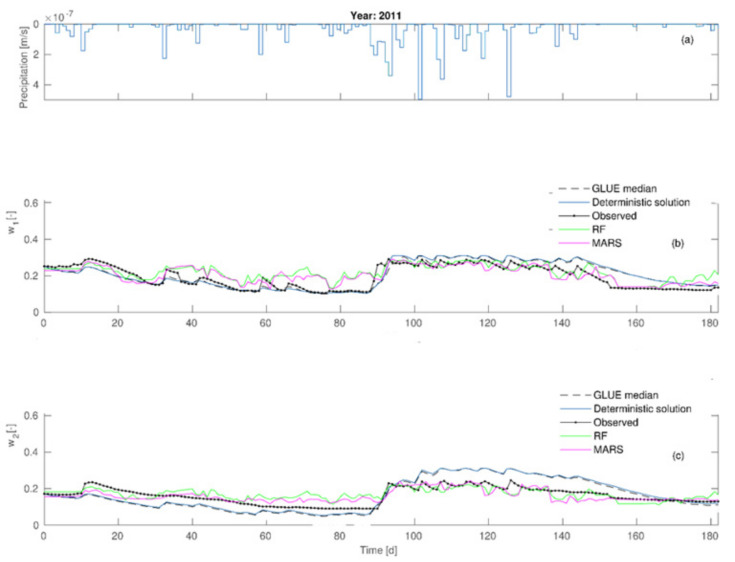
Comparison of the modeled and observed soil moisture contents by Deardorff model for the first calibration stage: (**a**) precipitation rates (**b**) *w*_1_-soil moisture content of the first layer (**c**) *w*_2_-soil moisture content of the second layer.

Taking advantage of a posteriori parameter distributions, the 95% confidence ranges were also determined for soil moisture contents *w*_1_ and *w*_2_ throughout the measurement period (Figure 5).

**Figure 6:**
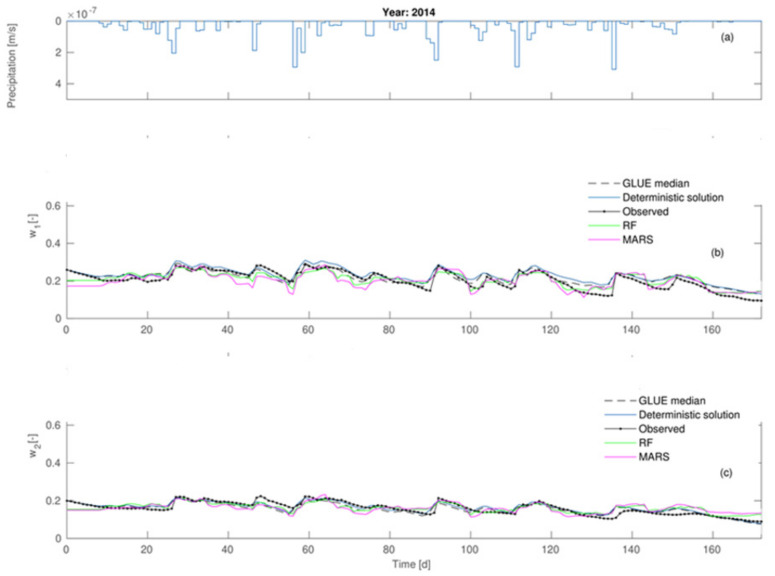
Comparison of the modeled and observed soil moisture contents by Deardorff model for the second calibration stage: (**a**) precipitation rates (**b**) *w*_1_-soil moisture content of the first layer (**c**) *w*_2_-soil moisture content of the second layer.

The data given in Figure 3 proves the most considerable influence of the C2 parameter on the agreement between the modeled and observed values. It is made evident by the highest values of the first order sensitivity (S_C2_ = 0.358) and the total sensitivity (S_TC2_ = 0.548) in comparison to other parameters’ sensitivities. Lesser influence on calibration results was shown by μ parameter which is supported by S_μ_ and S_T__μ_ values. Among all the analyzed parameters of the Deardorff model, the minor impact of *m* parameter was noticed. In this case, the values of S_i_ and S_Ti_ were equal to 0.019 and 0.168, respectively. Data shown in Figure 4 prove that the increase of p_1_ from 0 to 0.2 leads to the average value of reliability function range from 0.75 to 0.85. However, the case of p2 and p3 proves that the increase of those parameters’ values leads to decline of mean values of the reliability function and, moreover, the increase of p4 value considerably influences the agreement of the model and observations. It is also a fact that the increase from p3 = 0.02 to p3 = 0.08 caused the reliability function to range from 0.52 to 0.85. A considerable influence of ν parameter on reliability function was also noted, as the increase from ν = 2·10^−8^ to10^−7^ results in that function value to range from 0.02 to 0.93. Further increase of ν to 2·10^−7^ causes the decrease of reliability function value to 0.82. The existence of strong interactions between consecutive calibrated parameters (p1, p2, p3, p4, ν) is indicated by the achieved reliability function ranges. Calibration results of the Deardorff model (soil moisture content *w*_1_, *w*_2_) by the coupled GLUE-GSA method for the analyzed second period are given in Figure 5 and Figure 6. 

The results show *w*_1_ (average soil moisture content in layer1) to be underestimated by as much as 0.03 maximum in comparison to measured values for the period 0–85 days of the first calibration stage. However, the soil moisture content at 0.20 m (*w*_2_) was underestimated by 0.03 on average for the whole period of 0–85 days, where the daily average precipitation was equal to 1.64 mm and the total amounted to 143.1 mm. The next period, encompassing days 89–120, showed the moisture contents *w*_1_ and *w*_2_ to be overestimated in comparison with the measurements, and involved more intense daily average precipitation of 5.77 mm and a total of 369.3 mm. However, the period between day 120 and 180 exhibited *w*_1_ values to be overestimated by 0.08 maximum, and the observed difference between the model and observations decreased within the period, leading to their final equality. It was also noted that the simulated soil moisture content values in the second layer (*w*_2_) became lower than the observed ones after the 170th day. The second calibration phase (2014) was subject to considerably smaller errors. The achieved results pointed at predictive capabilities of the model to be dependent on dynamic meteorological conditions. This was the reason for the realization of additional soil moisture content simulations (*w*_1_, *w*_2_) aimed at detailed analyses of model predictive capabilities in order to select the optimum calculation method.

### 3.2. Statistical Models for Soil Moisture Content Forecast

Based on Section 2.3 and the presented relevant principles for model building by statistical methods BT, RF, MLP, MARS, and SVM, the simulations of soil moisture content were realized for both layers (*w*_1_, *w*_2_). The calculations involved numerous combinations of independent variables, describing precipitation totals (P), air temperature (T, T_sr_), and humidity (Wilg, Wilg_sr_). Table 3 and Table 4 present fitting quality (R, MAE, MAPE) of modeling results and observations for those combinations, achieved by BT, RF, MARS, MLP, GP, and SVM models. The lowest error values of moisture content simulations (*w*_1_, *w*_2_) were achieved by MARS model, in the following form:

*w*_1_ = 0.362 − 4.133*max(0; 4.87-Psr) + 0.257*max(0; 65.07-Wilgsr) + 2.182*max(0; Tsr-17.33) − 2.66*max(0; Psr-1.028) + 2.752*max(0; Psr-3.343) − 3.369*max(0; Tsr-19.97) − 1.157*max(0; Tsr-12.70) + 3.995*max(0; Tsr-21.671) (17)

*w*_2_ = 2.08 − 0.951*max(0; Psr-6.91) − 0.771*max(0; 6.914-Psr) − 0.174*max(0; 10.586-Tsr) + 0.132*max(0; 65.07-Wilgsr) + 2.369*max(0; Tsr-17.328) + 1.064*max(0; Psr-3.342) − 2.673*max(0; Tsr-19.38) − 1.148*max(0; Tsr-13.385) + 2.107*max(0; Tsr-21.071) (18)

In the case of BT and RF models, elaborated for *w*_1_ and *w*_2_ forecasting, the obtained number of trees ranged from 40 to 200, which means the models were not over-learned. For the MLP method the number of neurons ranged from 2 to 6, indicating that their maximum value (2·j + 1) was not exceeded. The proper activation functions in the hidden layer and the output layer were most frequently in the form of hyperbolic tangent and linear relationships. Moreover, the models achieved through the SVM method yielded the C parameter range 100–900 and α = 0.10–0.80, respectively.

All in all, the data given in Table 3 and Table 4 proved that precipitation, air temperature, and humidity influenced the soil moisture content most significantly. The smallest forecast errors for *w*_1_ and *w*_2_, taking into consideration only the precipitation totals, were obtained for average daily rainfall on a weekly basis. What is more, the achieved results point at more considerable impact of air temperature than humidity on soil moisture content, which is made evident by the estimated error values (MAE, MAPE). It points at a major influence of meteorological conditions which is also reflected by other research [33]. Among all analyzed methods, the smallest forecast errors of *w*_1_ and *w*_2_ were obtained by the MARS approach. More considerable errors were found for the SVM, BT and RF models. The analyzed combinations of independent variables provided the smallest error values of *w*_1_ and *w*_2_ forecasting for the P_sr_, Wilg_sr_, and T_sr_ (weekly averaged values) combination set.

All the above-mentioned models served to find the independent variables, which exhibited the best match of the modeled and observed values. Those variables seem to prove the soil moisture content to be conditioned by the dynamics of precipitation phenomena and meteorological conditions, first of all. Other impacts, such as soil type and its physical properties, may be found as important and cannot be omitted. Although these other impacts were not deeply considered herein, they deserve more insight in a future application of elaborated models. However, additional validation of these models in different precipitation and meteorological conditions should be treated as the most urgent issue.

### 3.3. Simulations of Soil Moisture Content by Deardorff and Statistical Models—Heat Maps

**Figure 7:**
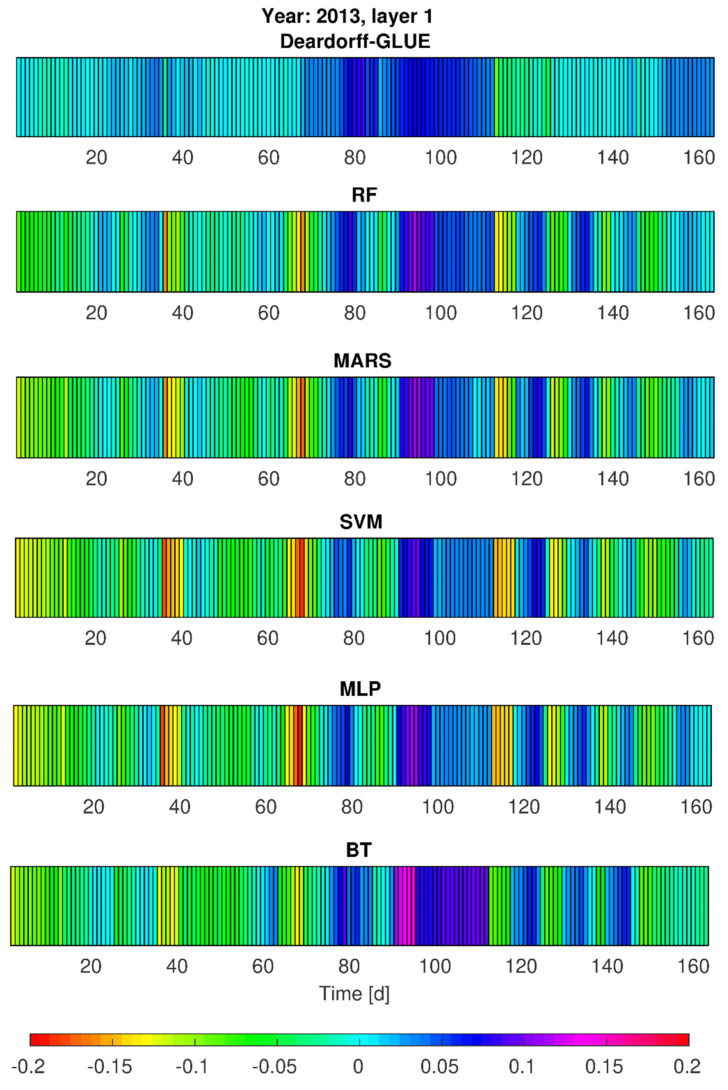
Error variability of soil moisture content forecast (*w*_1_, first layer) in 2013 by Deardorff model and statistical methods (BT, RF, SVM, MLP, MARS).

**Figure 8:**
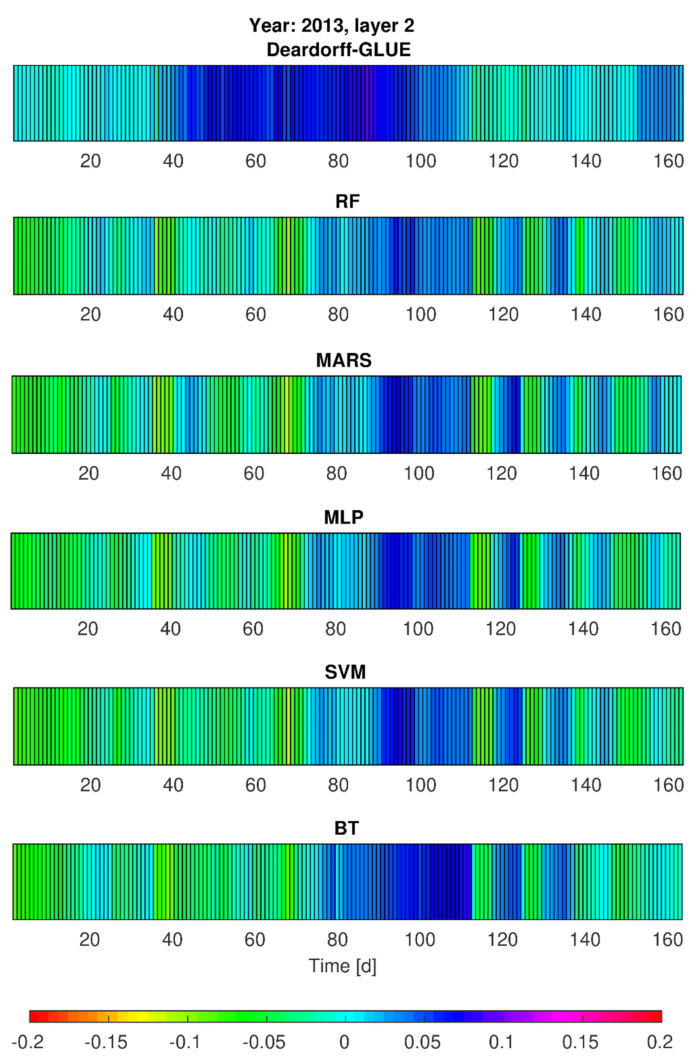
Error variability of soil moisture content forecast *w*_2_ (second layer) by Deardorff model and statistical methods (BT, RF, SVM, MLP, MARS) for 2013.

**Figure 9:**
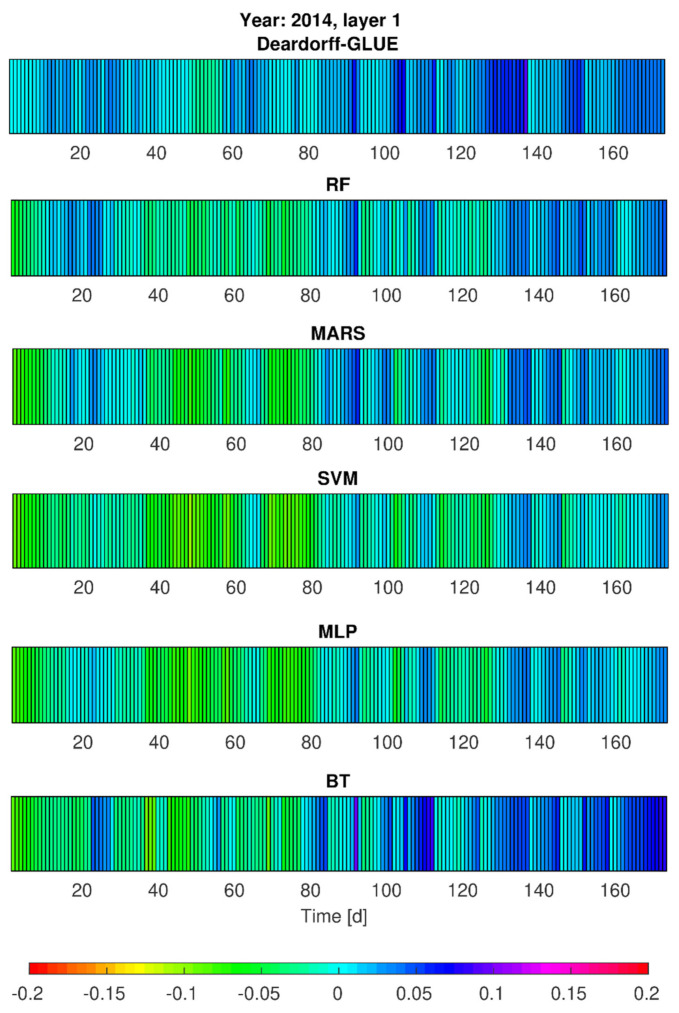
Error variability of the soil moisture content *w*_1_ (first layer) for 2014 by Deardorff model and statistical methods (BT, RF, SVM, MLP, MARS).

**Figure 10:**
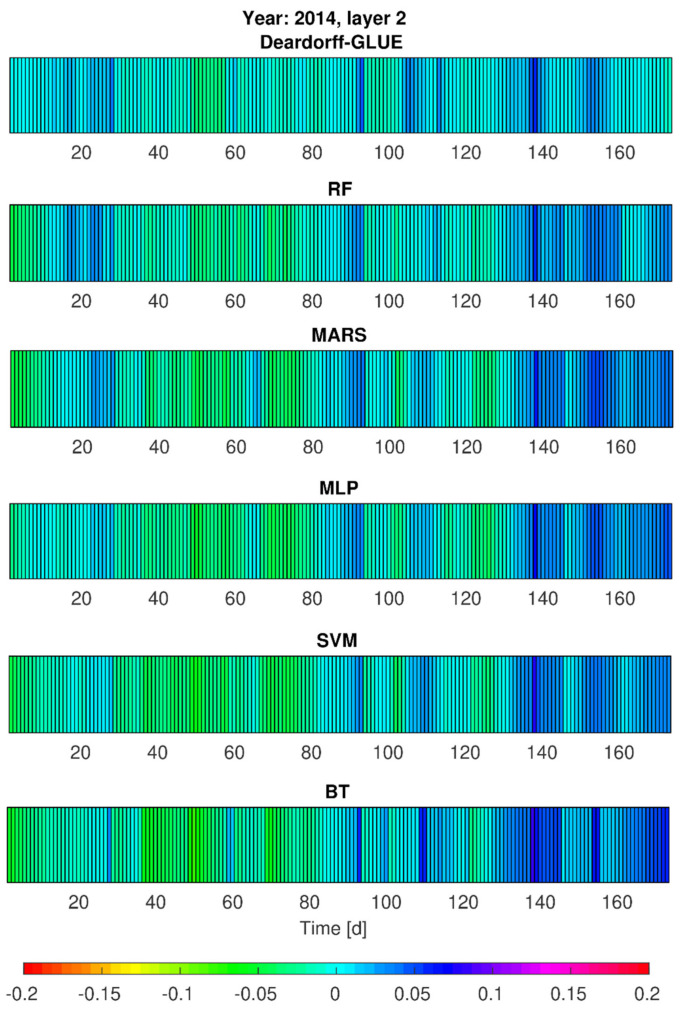
Error variability of the soil moisture content *w*_2_ (second layer) for 2014 by Deardorff model and statistical methods (BT, RF, SVM, MLP, MARS).

Taking into consideration the simulation results by statistical models on one hand, and the physical model results on the other hand, additional calculations of soil moisture content (*w*_1_, *w*_2_) were realized for vegetation seasons of 2013 and 2014 (calibration stages). This aimed to provide some insight into the models’ performance in slightly different development of hydrological and meteorological conditions (e.g., temporarily variable precipitation seasons). The achieved results, given in Figure 7, Figure 8, Figure 9 and Figure 10, prove that from all the applied methods (Deardorff model, BT, RF, MLP, SVM and MARS method) the best match of the modeled and the observed soil moisture contents was provided by Deardorff equation (physical one, Equations (4) and (5)). For most of the analyzed periods, the outcome of all applied models shows the overestimation of the simulated values in comparison to measurement data. This fact can be treated as a limitation in respect of the utilization of these models for soil moisture control. In realistic exploitation conditions, the soil moisture content would be lower than the mode result, which can lead to water shortages at the stage of decision making.

Based on the performed calculations by statistical models (RF, BT, SVM, MLP, MARS), and also by the Deardorff model, it was found that soil moisture contents (*w*_1_, *w*_2_) for no-rain periods were overestimated by 10% (SVM, MLP) or even by 15% (BT, RF, MARS, Figure 7 and Figure 8). The same tendency was revealed for 2014 in the second part of the analyzed period—mainly in September. The highest error values, exceeding 5%, were noticed for the BT method. Relatively small errors of *w*_1_ and *w*_2_ forecast were achieved by RF, BT, SVM, MLP, and MARS determined by the choice of independent variables. It was also noticeable that the simulated soil moisture content at the depth of 0.10 m (*w*_1_) in the case of intense precipitation (period between 30th and approximately 90th day) was locally lowered even by 20% (Figure 8) by the applied statistical models: MLP, SVM, MARS, and RF.

Simulation results of the soil moisture content for the depth of 0.20 m (*w*_2_), taking into account the single precipitation events, tended to be lower by about 5 to 10% in the adopted statistical models (Figure 7). In particular, this pertains to the period from the 30th to the 90th day for which the moisture contents were lower by as much as 5 to 10% in comparison with the observations (Figure 8).

Such values of the modeled soil moisture content result from the applied calculation procedures (models) and rely on the selection of the independent variables for the analyzed case study (P_sr_, Wilg_sr_, and T_sr_). Only the computations for MLP and MARS for 2013 showed that for single precipitation events the *w*_2_ values (moisture contents for the second layer) became overestimated by not more than 5%. However, for 2014, the period between the 30th and 90th day exhibited visibly lower simulated moisture contents for most of the precipitation events. On the other hand, taking into consideration such precipitation rates that are not of a more intensive character (e.g., the rates of *p* < (2–3)·10^−7^ m/s), the simulated values of the soil moisture content were underestimated by no more than 5%. Only in the case of lower precipitation intensity was a tendency of overestimated modeling results observed, especially for RF, MARS and SVM method (Figure 9 and Figure 10). Moreover, the application of elaborated statistical models through RF, BT, MLP, SVM, and MARS (Figure 7 and Figure 9) found that the simulated soil moisture contents *w*_1_ and *w*_2_ (both layers), preceded by a 23-day no-rain period in particular, were underestimated by about 15% maximum for SVM and MLP, 12% by MARS and RF, and 10% by BT. It was also noted, the second layer 0.20-m (*w*_2_) moisture contents were underestimated by as much as 5 to 10% by all the above-mentioned methods, relying on the choice of independent variables. It was found for the BT method, in particular, that for the period from July to August 2014 the soil moisture content was overestimated by 5 to 10%. Furthermore, the results provided by RF, SVM, MLP and MARS for the analyzed period, preceded by rainfall events of daily intensity ranging from (0.1–1)·10^−7^ m/s, tended to be overestimated by as much as 5%. In summary, the predictive behavior of all the elaborated statistical models in respect of the soil moisture contents seem to become strictly dependent on the character (particularly the actual values) of the input data (P_sr_, Wilg_sr_, and T_sr_).

## 4. Summary and Conclusions

The case of physically-based models results in more straightforward estimations of the consecutive, independent variables’ influence on the simulation results. It involves particular equations which make it possible to analyze and prove the impact of selected variables on the process dynamics. Statistical models, however, show more difficulties in this respect. All of the models that were approached in this work, as well as the coefficients describing their structure, encountered difficulties in their physical interpretation, but this was found only by this particular study and cannot be generalized. It should be mentioned that statistical models involved the interpolation of soil moisture contents on the basis of the data, which was utilized at the stage of learning and testing. The data used for calculations may involve identical or similar combinations of the independent variables which, in turn, influence the results of the forecasts. Physical models can avoid such problems. However, taking into account the fact that the Deardorff model overestimated the soil moisture contents, potential problems in decision-making may arise, affecting the determined goals that were suggested by this particular study, such as water reservoirs and hydraulic structures operation. It is based on the assumption that there is more water stored in the soil than there actually is, which can be considered a shortcoming in the management of water resources in a hypothetical area. This may lead to potential negative ecological and economic consequences from the viewpoint of decision-making and planning, therefore the need arises to introduce, for instance, safety factors. The value of that factor, assumed to be lower than one, would reduce the actual amount of water resulting from the simulations.

Nonetheless, the statistical models applied to TDR data (soil moisture contents) proved to have both a simple structure and interpretation character, showing considerable exploitation potential. The physically-based Deardorff model required additional assumptions on the formal relationships between the parameter values. Eventually, the measurement data gathered by TDR technique facilitated the uncertainty analysis on different types of models. Evidence was found for sufficient reproduction of the soil moisture contents for two layers (at 10 cm and 20 cm depth) of a loamy soil at a particular sampling point through a selection of statistical models and a physical one. Even if the proposed methods can be considered as universal, their future development and utilization requires calibration on a range of data coming from similar research sites.

## Figures and Tables

**Table 1:** Sensor types utilized for meteorological parameters monitoring.

Sensor Type	Parameter	Range	Error
Thermohygrometer Vaisala: QMH102 at the height of 2 m.	temperature	−40; +60 °C	<±0.3 °C
	relative humidity	0 to 90% RH	±2%
	relative humidity	90 to 100% RH	±3%
Sonic sensor Vaisala: WS425–B2A1B at the height of 4 m.	wind speed	0–56 m/s	±0.135 m/s or ±3% of the range
	wind direction	0–360°	±2° for wind speed > 2 m/s

**Table 2:** Assumed parameter ranges (*m*, *C*_2_, *μ*, *w_max_*).

Variables	Unit	Min	Max
*m*	-	0	2
*C*_2_	-	0	2
*μ*	-	0	10^−7^
*w_max_*	-	0.24	0.42

**Table 3:** Comparison of predictive capabilities for MARS, SVM, MLP models.

Parameter	First Layer						Second Layer					
	Learning			Testing			Learning			Testing		
	R	MAE	MAPE	R	MAE	MAPE	R	MAE	MAPE	R	MAE	MAPE
MARS												
P (t = 1)	0.60	4.00	20.58	0.43	5.10	31.20	0.58	2.79	18.14	0.50	3.21	22.77
P (t = 2)	0.58	3.82	19.95	0.51	4.37	29.04	0.62	2.73	17.95	0.52	3.08	22.05
P (t = 3)	0.60	3.66	19.37	0.51	4.34	26.66	0.60	2.64	17.58	0.55	2.95	21.15
P (t = 4)	0.46	3.52	18.87	0.57	3.92	24.16	0.59	2.64	17.78	0.58	2.78	20.09
P (t = 5)	0.63	3.38	18.05	0.61	3.67	22.63	0.60	2.62	17.59	0.62	2.63	19.10
P (t = 6)	0.66	3.30	17.49	0.65	3.41	21.13	0.62	2.55	17.07	0.65	2.50	18.24
P (t = 7)	0.67	3.20	16.90	0.66	3.31	20.41	0.66	2.48	16.63	0.67	2.39	17.50
P (me)	0.68	3.19	16.90	0.65	3.36	20.57	0.66	2.48	16.63	0.68	2.38	17.45
T (t−1)	0.60	3.89	20.04	0.39	5.45	33.19	0.61	2.69	17.55	0.48	3.31	23.27
Wilg (t−1)	0.60	4.03	20.64	0.38	5.58	34.14	0.62	2.73	17.68	0.47	3.35	23.55
P (me), T_sr_	0.81	2.48	12.05	0.81	2.50	15.90	0.79	1.93	12.43	0.74	2.15	15.53
P (me), Wilg_sr_	0.77	3.15	15.58	0.83	2.44	16.05	0.68	2.33	15.04	0.82	1.89	14.68
P (me), Wilg_sr_, T_sr_	0.85	2.45	12.03	0.92	2.07	12.22	0.82	1.96	12.62	0.94	1.55	11.28
MLP												
P (t = 1)	0.41	4.94	28.71	0.55	4.30	24.98	0.39	3.02	20.69	0.34	3.51	27.94
P (t = 2)	0.53	4.57	26.63	0.52	4.37	24.59	0.42	3.16	22.94	0.52	3.13	22.23
P (t = 3)	0.60	3.94	23.18	0.61	3.95	22.90	0.50	2.99	22.14	0.58	2.86	20.44
P (t = 4)	0.67	3.46	20.03	0.71	3.64	20.08	0.58	2.39	16.71	0.59	2.86	21.16
P (t = 5)	0.69	3.46	19.83	0.71	3.20	17.93	0.61	2.63	17.72	0.65	2.81	22.20
P (t = 6)	0.76	2.84	16.76	0.72	3.00	17.00	0.66	2.16	15.53	0.63	2.79	20.53
P (t = 7)	0.69	3.20	19.03	0.77	2.92	16.50	0.62	2.66	20.34	0.71	2.19	14.11
P (me)	0.75	2.99	17.33	0.76	2.85	16.39	0.68	2.40	18.02	0.69	2.37	18.10
T (t−1)	0.14	5.38	31.86	0.18	5.38	28.98	0.17	3.33	23.06	0.20	3.40	24.29
Wilg (t−1)	0.21	5.20	30.09	0.29	5.59	33.47	0.28	3.38	23.26	0.17	4.17	30.34
P (me), T_sr_	0.81	2.54	16.90	0.80	2.71	15.50	0.65	2.56	18.47	0.73	2.37	17.24
P (me), Wilg_sr_	0.76	2.89	17.17	0.83	2.33	13.18	0.65	2.24	16.33	0.65	2.31	18.07
P (me), Wilg_sr_, T_sr_	0.82	2.50	16.18	0.84	2.15	15.50	0.67	2.18	15.72	0.68	2.20	16.00
SVM												
P (t = 1)	0.43	4.57	23.43	0.31	4.75	26.12	0.33	3.11	21.38	0.13	3.08	21.53
P (t = 2)	0.51	4.22	21.62	0.54	4.01	22.08	0.39	2.99	20.87	0.39	2.77	19.65
P (t = 3)	0.56	3.89	20.18	0.68	3.45	19.52	0.45	2.87	20.50	0.52	2.57	18.63
P (t = 4)	0.62	3.62	19.34	0.76	2.92	17.24	0.49	2.78	20.29	0.61	2.37	17.83
P (t = 5)	0.67	3.32	18.01	0.74	3.05	18.02	0.56	2.62	19.34	0.56	2.42	18.21
P (t = 6)	0.71	3.13	17.15	0.73	2.96	17.63	0.61	2.51	18.55	0.59	2.32	17.81
P (t = 7)	0.73	2.98	16.35	0.72	2.99	17.53	0.65	2.41	17.68	0.61	2.29	17.16
P (me)	0.74	2.92	16.35	0.73	2.95	17.05	0.68	2.32	17.15	0.62	2.26	16.56
T (t−1)	0.20	5.14	28.43	0.18	5.74	33.92	0.19	3.26	23.50	0.13	3.28	24.62
Wilg (t−1)	0.19	5.30	30.56	0.18	5.38	33.14	0.07	3.42	22.46	0.02	3.09	21.60
P (me), T_sr_	0.78	2.86	15.15	0.79	2.56	14.26	0.67	2.34	17.61	0.62	2.33	16.70
P (me), Wilg_sr_	0.76	2.86	15.79	0.76	2.86	16.11	0.72	2.21	16.47	0.67	2.11	15.31
P (me), Wilg_sr_, T_sr_	0.83	2.42	13.74	0.84	2.39	13.47	0.67	2.35	17.46	0.64	2.16	15.85

**Table 4:** Comparison of predictive capabilities for BT, RF models.

Parameter	First Layer						Second Layer					
	Learning			Testing			Learning			Testing		
	R	MAE	MAPE	R	MAE	MAPE	R	MAE	MAPE	R	MAE	MAPE
BT												
P (t = 1)	0.49	4.63	27.64	0.33	5.13	27.93	0.47	2.99	21.66	0.27	3.63	23.33
P (t = 2)	0.59	4.09	24.43	0.52	4.43	24.15	0.55	2.83	20.54	0.48	3.32	21.50
P (t = 3)	0.62	3.92	22.85	0.55	3.93	21.91	0.56	2.71	19.17	0.50	2.83	18.62
P (t = 4)	0.69	3.39	19.49	0.65	3.44	18.71	0.63	2.51	18.06	0.62	2.48	16.44
P (t = 5)	0.74	3.08	17.58	0.71	3.00	16.31	0.64	2.48	17.85	0.61	2.56	17.17
P (t = 6)	0.74	3.10	17.83	0.78	2.71	15.34	0.66	2.40	17.05	0.72	2.25	15.26
P (t = 7)	0.78	2.82	16.21	0.79	2.66	15.11	0.67	2.30	16.34	0.75	2.14	14.56
P (me)	0.78	2.85	16.40	0.80	2.67	14.50	0.69	2.24	15.82	0.78	2.01	13.94
T (t−1)	0.22	5.29	30.70	0.26	5.09	28.43	0.42	3.05	21.19	0.31	3.02	19.87
Wilg (t−1)	0.25	5.31	30.10	0.12	5.32	29.43	0.29	3.31	22.39	0.10	3.48	22.29
P (me), T_sr_	0.81	2.66	15.51	0.80	2.80	15.68	0.71	2.20	15.66	0.72	2.33	15.61
P (me), Wilg_sr_	0.79	2.79	16.07	0.80	2.81	15.42	0.71	2.23	15.99	0.70	2.25	14.78
P (me), Wilg_sr_, T_sr_	0.81	2.68	15.36	0.81	2.72	14.71	0.73	2.15	15.09	0.69	2.20	14.00
RF												
P (t = 1)	0.50	4.66	27.72	0.35	4.89	27.13	0.40	3.16	22.38	0.12	3.62	24.96
P (t = 2)	0.61	4.11	24.25	0.41	4.72	26.23	0.57	2.77	19.43	0.12	3.77	26.27
P (t = 3)	0.63	3.76	21.95	0.59	3.97	22.70	0.58	2.58	18.45	0.38	3.02	21.12
P (t = 4)	0.69	3.37	19.94	0.70	3.39	19.60	0.62	2.47	17.79	0.57	2.61	18.43
P (t = 5)	0.75	2.92	17.38	0.72	3.17	18.15	0.67	2.28	16.66	0.63	2.53	17.95
P (t = 6)	0.77	2.83	17.05	0.75	2.98	16.68	0.71	2.15	15.94	0.59	2.65	18.51
P (t = 7)	0.81	2.54	15.28	0.77	2.86	16.34	0.74	1.98	14.61	0.60	2.64	18.23
P (me)	0.83	2.34	14.03	0.78	2.79	14.94	0.74	2.04	14.59	0.61	2.48	17.07
T (t−1)	0.37	4.83	28.59	0.06	5.38	30.42	0.36	3.03	21.31	0.20	3.38	23.06
Wilg (t−1)	0.32	5.13	29.99	0.18	5.49	30.50	0.32	3.27	22.44	0.06	3.55	24.20
P (me), T_sr_	0.87	2.72	16.34	0.75	3.30	18.66	0.82	2.06	14.78	0.59	2.67	19.14
P (me), Wilg_sr_	0.85	2.96	17.66	0.80	3.30	18.89	0.80	2.05	14.92	0.58	2.70	19.50
P (me), Wilg_sr_, T_sr_	0.86	2.93	17.40	0.83	2.96	17.80	0.78	2.01	14.50	0.68	2.20	15.20

## Data Availability

Not applicable.
